# Clinical Remarks upon Surgical Cases in the Buffalo General Hospital—Amputation of the Cervix Uteri

**Published:** 1868-11

**Authors:** J. F. Miner


ART. III.—*Clinical Remarks upon Surgical Cases in the Buffalo General Hospital—Amputation of the Cervix Uteri.* BY J. F. MINER, M. D.

GENTLEMEN:—I am most happy to introduce you this morning to the new operating amphitheatre provided for your accommodation in the Buffalo General Hospital, and making myself your representative, allow me to express in your behalf as well as on my own, many hearty thanks to the Trustees and Superintendent, Mr. Bagley, for the opportunities they afford the Faculty of the University of providing increased facilities for clinical instruction. As now provided, no institution in the country can offer better opportunities in this respect; the material is abundant, and above all is from the fields of private practice, and not of the ordinary hospital variety. Many of the cases come in hither from the families of the city or country, for operation or advice, by suggestion of myself, my associates, or some professional friends; they are mostly such cases as you will meet in private practice, and are valuable on this account as well as from the intrinsic interest they possess. It is also common to present in this institution some of the most remarkable forms of disease, affording important and rare surgical operations. Our first operation this morning is of this description, and is offering you an opportunity which medical students hardly ever enjoy.

Our patient is an unmarried, highly respectable lady, 29 years old, and in all respects healthy, except that she suffers from hypertrophy of the cervix uteri; the uterine neck is elongated, so that it is at least three inches in length. It is not greatly enlarged in its transvere diameter, neither is the body of the uterus appreciably increased in size or weight. When standing, the os uteri protrudes from the vagina, when recumbent is just within the orifice of vulva. The general disturbance is severe—the menstrual period is prolonged and painful, and the secretion profuse. Walking is difficult, almost impossible. Various nervous disturbances are almost always present, though the general appearance of health has been quite well preserved. The case is one of *simple uterine hypertrophy*, the enlargement limited to the neck of the womb. The ailment seems to consist of simple overgrowth of the part, the neck of the womb being in all respects healthy to the touch, and free from all disease, except that induced by irritation from its exposed situation. The chief increase is in the length of the uterine neck, giving rise to the symptoms of *prolapsus*—indeed this disease is often taken for it by patient or by the physician who makes hasty and superficial examination. This condition presents mechanical impediment to sexual intercourse, might induce sterility, and does give rise to all the most distressing symptoms of prolapsus—weight and bearing down pains in the pelvis increased at each menstrual period and usually aggravated by exertion.

The causes of such disease are not wholly apparent. *Disordered nutrition* is a pathological phrase to denote such change, and is said to arise from general and local causes. We do not understand very much about the changes in nutrition by which the whole system sometimes becomes greatly increased in size and weight, so that an apparently healthy person grows from one hundred fifty pounds to six or seven hundred, mostly by accumulation of fat. We observe that *chronic inflammation* sometimes induces increase in size of organs or parts of organs, and it is regarded as one of the causes of hypertrophy. *Increase of action or effort* induces increase of size, as is so often seen in the heart from valvular insufficiency, and is a second cause of hypertrophy. The same is observed elsewhere in the system, perhaps everywhere, increased action and effort is attended by increased strength, or size, or weight, or all of these combined. The history of this patient does not furnish any hint as to causes which may have operated to produce this unnatural development of the uterine neck, and all speculations as to the causes of hypertrophy of this or other organs in general, leads to no valuable conclusions in the case before us.

There can be no cure of this affection, except the removal of the superfluous growth, and when it has reached so great an elongation as this, there can be no doubt as to the propriety of removal; the smaller degrees of hypertrophy might perhaps very well be left without interference. Authors inform us that this operation is by no means devoid of risk, and that the danger arises from hemorrhage, which it has been found impossible to control, metritis, peritonitis, and from all the other common sources of danger after surgical operations. Improved methods of operation have lessened the dangers, so that it is now generally regarded as more safe and successful in its results.

It is proposed to remove the entire neck, and as the patient is now fully under the influence of chloroform you will observe the manner of operation. I have grasped the lower portion with hooked forceps, and by a very little traction the whole neck of the organ is fully exposed. With strong scissors we now proceed to divide it, making a flap in the same manner and shape we would do in the amputation of a finger or of any of the extremities. After completing the upper or anterior flap a suture is passed on both sides a little from the centre—a little way from the os as it will be when healed; now one-half of the remaining posterior flap is made and the suture passed through it, lastly, enough of the remaining part is divided to afford room for the passage of the other suture, which is also introduced before the whole is severed, thus the wound has been closed as soon as made—I was about to say *before* it was made. By this means we have avoided all injury of the organ by introduction of hooks or forceps to maintain it in exposed position, and when wholly divided nothing has remained but to tie the sutures securely. All hemorrhage has been avoided; perfect control of the organ has been preserved until the operation is complete and there has been no injury inflicted upon it in efforts to preserve it external to the vulva. This plan of making a flap and closing the wound, is perhaps due to the invention of Dr. Sims; this mode of introducing the sutures as we go along is an invention of my own, and made this morning to suit the exigencies of this case. If it has been practiced by surgeons before, I am glad of it, for I like it very much, and believe they must have operated better if they instituted this method; I shall not contend for the honor of the invention. Various modes have been practiced for amputation of the uterine neck which has been instituted largely for removal of malignant disease. It has been practiced in almost all countries with most signal and uniform failure. Malignant disease of the uterus may have been delayed in its progress by removal, but if any cases are reported as permanently cured, it is vastly more probable that they were not malignant, and that removal was made of simple and benign growths. The earlier amputations were made with the knife and hemorrhage was exceedingly troublesome, sometimes dangerous and even fatal. Later the eccraseur was used, an instrument by which the neck was severed slowly and the parts crushed off rather than cut. The instrument is on our table and you observe the manner of its working. By this method the danger of hemorrhage was greatly lessened, but a wide, open wound was left, requiring a long time for granulation, and offering an attractive field for fungus growths, the very disease for removal of which, the operation was most frequently made. In many instances, however, the operation can be made with this instrument, and can hardly be effected by any other plan. The operation, however, which you have observed when possible, offers vast improvements, and for removal of conditions such as was present with the young woman this morning, cannot be difficult or in any way very dangerous, and I anticipate for it a most satisfactory result.

